# Examine Your Medicines

**Published:** 1858-08

**Authors:** Jno T. Plummer


ARTICLE V.

### EXAMINE YOUR MEDICINES.

TO THE EDITORS OF THE CHICAGO MEDICAL JOURNAL,—Permit a stranger to your pages (a stranger, not as a reader, but a writer), to call the attention of your readers very succinctly to the necessity of increased care in the choice and administration of our medicines.

Beside the ordinary adulterations with which we are all familiar, there appears to have been recent developments of new and dangerous additions to our common remedial agents which claim our serious notice. Thus, in the "Transactions of the College of Physicians of Philadelphia," we find Dr. Keating relating a case of an alarming character from the administration of three grs. of what purported to be Tilden's extract of hyoscyamus. The dose was followed by morbid respiration, complete insensibility, small and rapid pulse (150 in a minute), profuse, clammy sweat, pupils extremely dilated, scarlet effusion of the skin and inability to swallow. Dr. Ruschenberger corroborated the case by another in his own practice, in which a sailor, by the use of four grs. of similar extract, became delirious, had dilated pupils, troubled vision, red skin and dryness of the fauces. The reporter remarks that "the general impression" of the members present "seemed to be that the symptoms described were those of belladonna only, and that, as the extract employed had not been tested by analysis, there must have been more or less extract of belladonna."

To these cases I may add one of my own. A little child took, in a compound of calomel, ipecac and prepared chalk for dysentery, one-fourth of a grain of soft extract of hyoscyamus; in an hour or two, its skin became red, its fauces dry, eyes affected, convulsive motions of its arms, eyelids and head, etc. This occurring but a day or two since, I have not examined the extract for belladonna; which, however, I fear, will be detected in the preparation.

At the same meeting of the college, Dr. Roger states that he was induced to examine ten specimens of sub-nitrate of bismuth from different apothecaries in Philadelphia, from the occurrence of a fatal case from the use of this powder, and that he detected arsenic in eight of the samples. This dangerous adulteration is not altogether new; but it has rarely been suspected, and as rarely looked for. All our authorities agree in representing earthy carbonates and carbonate of lead as the more common admixtures; but according to Prof. Bache (U. S. Disp.,) Lassaigne obtained one-sixth of one per cent. of arsenic from a parcel of sub-nitrate of bismuth bought in Paris.

Again, one of our apothecaries sold some sugar-coated compound calomel pills, which, the physician says, acted so drastically as to excite inquiry into the cause. Tilden was written to, and replied that the pills returned had been analyzed and found correct. Notwithstanding this, I subjected some of the same pills to a careful examination, and the result was, that I found the guaiacum, calomel, etc., all present, but not a particle of sulphuret of antimony; in the place of it, I obtained unequivocal evidence of the presence of tartarized antimony, by its reaction with metallic zinc, sulphuric acid, etc.

The subject is one which claims a more deliberate consideration than I am now able to bestow upon it, and therefore I submit the foregoing hastily penned paragraphs as simply suggestive.

JNO. T. PLUMMER.

